# Solution Structures and Dynamic Assembly of the 24-Meric Plasmodial Pdx1–Pdx2 Complex

**DOI:** 10.3390/ijms21175971

**Published:** 2020-08-19

**Authors:** Najeeb Ullah, Hina Andaleeb, Celestin Nzanzu Mudogo, Sven Falke, Christian Betzel, Carsten Wrenger

**Affiliations:** 1 Institute of Biochemistry and Molecular Biology, Laboratory for Structural Biology of Infection and Inflammation, University of Hamburg, c/o DESY, Build. 22a. Notkestr. 85, 22603 Hamburg, Germany; najeeb.ullah@chemie.uni-hamburg.de (N.U.); hina.andaleeb@chemie.uni-hamburg.de (H.A.); cmudogo@gmail.com (C.N.M.); falke@chemie.uni-hamburg.de (S.F.); 2 Department of Biochemistry, Bahauddin Zakariya University, Multan-60800, Punjab, Pakistan; 3 Department of Basic Sciences, School of Medicine, University of Kinshasa, Kinshasa BP834 KinXI, Congo; 4 The Hamburg Centre for Ultrafast Imaging (CUI), Luruper Chaussee 149, 22761 Hamburg, Germany; 5 Unit for Drug Discovery, Department of Parasitology, Institute of Biomedical Sciences, University of São Paulo, Av. Prof. Lineu Prestes 1374, 05508-000 São Paulo-SP, Brazil

**Keywords:** pyridoxal phosphate (PLP) synthase, Pdx1 and Pdx2, solution structure, protein–protein interactions, reversible oligomerization, drug discovery, malaria parasites, vitamin B6 synthesis

## Abstract

*Plasmodium* species are protozoan parasites causing the deadly malaria disease. They have developed effective resistance mechanisms against most antimalarial medication, causing an urgent need to identify new antimalarial drug targets. Ideally, new drugs would be generated to specifically target the parasite with minimal or no toxicity to humans, requiring these drug targets to be distinctly different from the host’s metabolic processes or even absent in the host. In this context, the essential presence of vitamin B_6_ biosynthesis enzymes in *Plasmodium*, the pyridoxal phosphate (PLP) biosynthesis enzyme complex, and its absence in humans is recognized as a potential drug target. To characterize the PLP enzyme complex in terms of initial drug discovery investigations, we performed structural analysis of the *Plasmodium viva*x PLP synthase domain (Pdx1), glutaminase domain (Pdx2), and Pdx1–Pdx2 (Pdx) complex (PLP synthase complex) by utilizing complementary bioanalytical techniques, such as dynamic light scattering (DLS), X-ray solution scattering (SAXS), and electron microscopy (EM). Our investigations revealed a dodecameric Pdx1 and a monodispersed Pdx complex. Pdx2 was identified in monomeric and in different oligomeric states in solution. Interestingly, mixing oligomeric and polydisperse Pdx2 with dodecameric monodisperse Pdx1 resulted in a monodispersed Pdx complex. SAXS measurements revealed the low-resolution dodecameric structure of Pdx1, different oligomeric structures for Pdx2, and a ring-shaped dodecameric Pdx1 decorated with Pdx2, forming a heteromeric 24-meric Pdx complex.

## 1. Introduction

Malaria, with more than 250 million humans infected annually and up to 0.5 million fatalities [1], highlights an urgent need to identify and discover new antimalarial drugs [2] for use against the human pathogens *Plasmodium falciparum, P. vivax, Plasmodium knowlesi, Plasmodium malariae,* and *Plasmodium ovale,* in particular [2,3]. Drug targets that interfere with the metabolism of the parasite, like the vital vitamin pathways, are the focus of the latest drug discovery investigations [4]. Although *P. falciparum* is responsible for the majority of the cases and deaths, *P. vivax* has a wider geographical distribution and is the causative organism of almost half of malaria cases outside Africa. Further, it generates re-emerging quiescent liver-stage parasites, causing repeated clinical episodes of malaria due to a single infection. Therefore, the World Health Organization (WHO) currently prioritizes investigations targeting *P. vivax* rather than other *Plasmodium* species [5,6].

Pyridoxal-5-phosphate (PLP), the active cofactor for more than 100 vitamin B_6_-dependent enzymes, results from the transformation of its precursors, namely pyridoxine, pyridoxamine, and pyridoxal. [7,8]. Bacteria, fungi, and plants synthesize PLP de novo via the vitamin B_6_ biosynthesis pathway, whereas mammals are entirely dependent on its uptake [9]. Biochemical analysis unveiled that the PLP synthase complex functions as a glutamine amidotransferase to produce PLP, where Pdx2 functions as the glutaminase domain to metabolize glutamine to ammonia and Pdx1 as the synthase domain, which utilizes the substrates ribose-5-phosphate, glyceraldehyde-3-phosphate, and ammonia [10,11]. The ammonia produced by Pdx2 is supplied to the active site of the synthase domain (Pdx1) through a hydrophobic ammonia tunnel [12].

Biochemical and biophysical characterizations of *P. falciparum* and *Bacillus subtilis* PLP synthases revealed that eukaryotic and prokaryotic Pdx complexes have substantial structural differences [12,13]. The crystal structures of Pdx1 from *Geobacillus stearothermophilus* (4WXZ), *B. subtilis* (2NV1), [14,15], and *Mycobacterium tuberculosis* (4JDY) [16] unveiled a dodecameric structure. However, for *Saccharomyces cerevisiae* Pdx1, analytical ultracentrifugation (AUC) and X-ray crystallography studies revealed a hexamer (3FEM) [17]. Crystal structures of Pdx2 (1R9G, 2ABW) [18,19], and the PLP synthase complex were reported for prokaryotes with protein data bank (pdb) codes 2NV2, 2ISS, 4WXY [15,20,21] and for a *Plasmodium* chimeric complex (4ADS) [22]. The Pdx complex is more stable in *Plasmodium* than in *Bacillus* [12], and Pdx1 and the Pdx1–Pdx2 complex are substantially different in terms of oligomerization and stability in solution [12,22].

Knowing the differences between eukaryotic and prokaryotic Pdx1 and Pdx complexes and gaining insight into plasmodial Pdx1 and Pdx complex dynamic oligomerization behaviors in solution would support drug discovery targeting the Pdx complex. Therefore, we investigated *P. vivax* Pdx1 and Pdx2 and the Pdx complex by applying complementary bioanalytical techniques. The data obtained by applying dynamic light scattering (DLS), small angle X-rays scattering (SAXS), and electron microscopy (EM) provide insights into the structure, dynamics, and oligomerization behaviors of Pdx1 and Pdx2 and their interactions in solution.

## 2. Results

### 2.1. Expression and Purification

**Figure 1:**
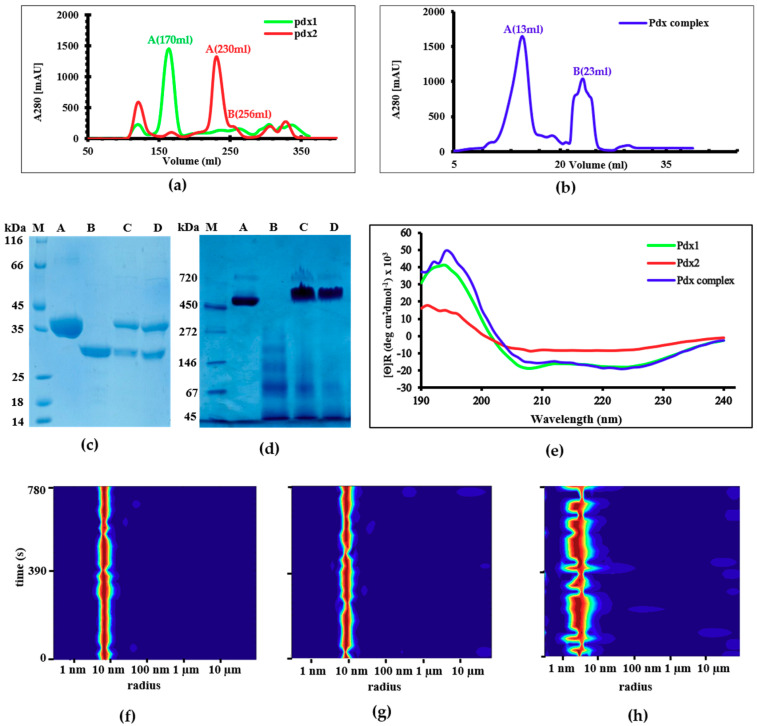
Size-exclusion chromatography (SEC) elution profile of (**a**) Pdx1 and Pdx2 using a Superdex 200 26/60 column and (**b**) the Pdx complex using a Superose-6-increase 10/300 column; (**c**) SDS-PAGE of the purified samples: lane A corresponds to Pdx1, lane B to Pdx2, lane C to the Pdx1–Pdx2 complex before SEC, and D shows the SEC-purified Pdx complex; (**d**) Native PAGE showing the unreduced form of the Pdx proteins: lane A: Pdx1 dodecamer; lane B: Pdx2 in different oligomeric states; lane C: a Pdx1 and Pdx2 sample mixed in 1:1 molar ratio applied to SEC; lane D: purified Pdx complex at approximately 700 kDa; (**e**) Circular Dichroism (CD) spectroscopy results showing secondary structure contents of Pdx1 (green), the complex (blue) with α-helices and β-sheets as predominant secondary structure elements, and Pdx2 (red) with α-helices, β-sheets, and some random coils. Experimental CD data obtained for *Pv*Pdx2 were compared with different secondary structure content predicting servers, i.e., SOMPA (α-helices 33, β-sheets 21, and coils 40), DSSP (α-helices 29, β-sheets 32, and coils 39), and Predict Protein (α-helices 29, β-sheets 27, and coils 44). Calculated data are consistent with the experimental data. Dynamic light scattering (DLS) analysis of (**f**) dodecameric Pdx1 (R_H_ = 7.3 ± 0.9 nm), (**g**) monodispersed saturated Pdx1–Pdx2 complex (R_H_ = 9.7 ± 0.2 nm), and (**h**) monomeric Pdx2 (R_H_ = 2.8 ± 0.4 nm).

*Pdx1* and *Pdx2* genes were successfully overexpressed and corresponding active proteins with molecular weights of 35 and 26 kDa, respectively, were purified using affinity and size-exclusion chromatography (SEC). Using SEC, Pdx1 was eluted at 170 mL (Figure 1a). The elution profile for Pdx1, obtained by applying a Superdex S200 26/60 column for purification, is shown in Figure 1a (green). The protein fraction collected from corresponding peak A was evaluated by SDS-PAGE (Figure 1c,d, lane A), indicating a dodecamer in the solution. Pdx2 was eluted at 230–256 mL (Figure 1a, red), with its corresponding peaks A and B evaluated by PAGE (Figure 1c,d, lane B) and a monomer and different oligomers indicated in solution. Pdx1 and Pdx2 were mixed in a 1:1 molar ratio and applied to a Superose-6-increase column. The elution profile is shown in Figure 1b, with peak A corresponding to the *Pv*Pdx1/*Pv*Pdx2 complex, confirmed via SDS-PAGE (Figure 1c, lane D). The Pdx1–Pdx2 complex was eluted at approximately 15 mL, with a corresponding molecular weight of approximately 700 kDa (Figure 1d, lane D). This chromatography fraction was assessed via SDS-PAGE, showing two bands at 35 and 26 kDa corresponding to reduced monomeric Pdx1 and Pdx2 (Figure 1c). The purified Pdx complex was further characterized for functionality according to [7], and by native PAGE, a band corresponding to a molecular weight of 650–700 kDa (Figure 1d) was observed.

### 2.2. Circular Dichroism (CD) Spectroscopy and DLS Investigations

Secondary structure information obtained by applying CD spectroscopy indicated 52% α-helix and 20% β-sheet contents for Pdx1, 36% and 34% for Pdx2, respectively, and 54% and 25% for the complex, respectively, with corresponding mean RMS (root mean square) values of 8.4, 6.2, and 6.5, respectively, using the JASCO Spectra Manager software suite for data evaluation (Figure 1e) [23]. The CD data and corresponding secondary structure contents were compared with the secondary structure content of the homologous *Pf*Pdx2 crystal structure (pdb code: 2ABW) [19]. Further, the *Pv*Pdx2 secondary structure content was calculated by applying different secondary structure prediction servers, i.e., SOPMA (self-optimized prediction method with alignment), DSSP (define secondary structure of proteins), and Predict Protein. The resulting values are described in the legend of Figure 1e.

Dynamic light scattering is a useful technique to investigate the size distribution and dispersity of nanoparticles or macromolecular complexes in solution [24,25]. For the monodispersed *P. viva*x Pdx1 solution, DLS showed a hydrodynamic radius of R_H_ = 7.3 ± 0.9 nm (Figure 1f), confirming its dodecameric state in solution [22]. For the Pdx complex, a hydrodynamic radius of 9.7 ± 0.2 nm was observed (Figure 1g). For Pdx2, DLS experiments revealed a concentration and time-dependent oligomerization tendency, with resulting R_H_ values of 2.8 ± 0.4 nm at 1.0 mg mL^−1^ concentration, corresponding to approximately 35 kDa (Figure 1h) and 3.5 ± 0.4 nm at 1.6 mg mL^−1^ and 4.2 ± 0.4 nm at 2.5 mg, corresponding to approximately 60 kDa and 90 kDa, respectively (Supplementary Figure S1a,b). Most interestingly, mixing an oligomeric Pdx2 suspension, which was shown via DLS to have a multiple radius distribution (Supplementary Figure S1a–d) with dodecameric monodispersed Pdx1, resulted in a monodispersed Pdx complex with a hydrodynamic radius of 10.9 ± 1.4 nm (Supplementary Figure S1e).

### 2.3. Reversible Oligomerization of Pdx2 by DLS Investigation

We analyzed the reversible oligomerization tendency of Pdx2 by applying time-resolved DLS and mixing nonhomogeneous Pdx2 oligomers with homogenous dodecameric Pdx1. A Pdx complex formation was observed by utilizing dodecameric Pdx1 and Pdx2 (monomeric and oligomeric) in independent experiments. The plots in the center display the respective size distribution over time. The abundance of radii (number of particles scattering the laser light) is color-coded from blue (low) to red (high).

**Figure 2:**
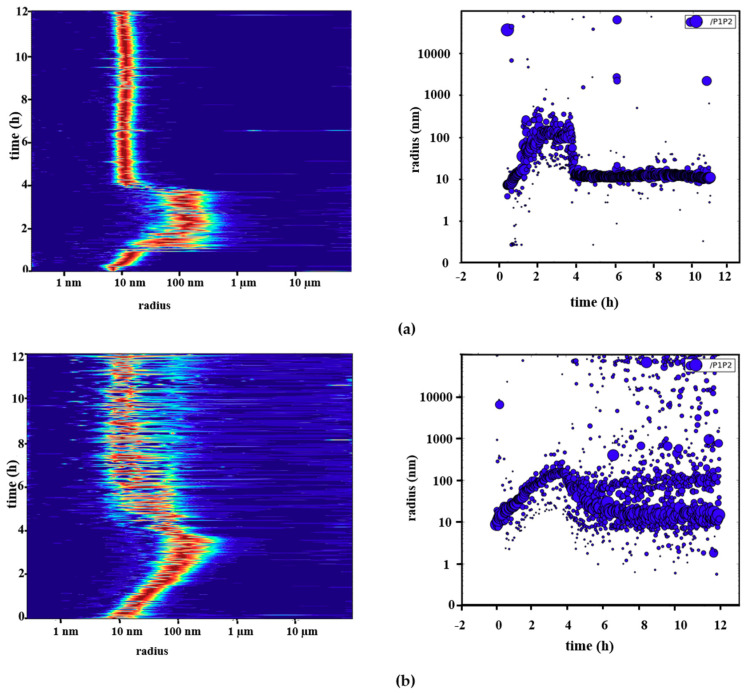
Figures on the left in red indicate the particle size distribution, with the y-axis corresponding to the time course of the experiment and the horizontal axis showing the radius distribution. For figures on the right side, the axes are inverse. (**a**) Particle size distribution of dodecameric Pdx1 mixed with monomeric Pdx2 measured over 12 h after applying DLS. First, dodecameric Pdx1 was measured for 25 min after a solution of Pdx2 was added. After mixing Pdx1 and Pdx2, the predominant particle radii distribution shifted toward larger radii of approximately 100–300 nm. The high-molecular-weight particles (R_H_ > 100 nm) remained for two to three hours. Subsequently, the particle radii shifted to approximately 10–14 nm, corresponding to the radius of the Pdx complex; (**b**) DLS measurements of a suspension containing dodecameric Pdx1 and oligomeric Pdx2 recorded over time. After 15 initial measurements of dodecameric Pdx1, oligomeric Pdx2 was added and the radius profile shifted toward higher radius values, i.e., oligomeric Pdx2 interacted with Pdx1 to form the Pdx complex. After 4–5 h, the equilibrium shifted toward a radius corresponding to the Pdx complex radius with some remaining polydispersity. As seen from the radius profile, some large oligomeric particles, presumably corresponding to Pdx2 oligomers, remained in solution.

After mixing dodecameric Pdx1 and monomeric Pdx2 in a 1:1 molar ratio, interactions between the two proteins were gradually established, resulting in a monodispersed Pdx complex at a concentration of 5 mg mL^−1^ with R_H_ = 10.4 nm (Figure 2a and Supplementary Video S1). By mixing a nonhomogeneous solution of oligomeric Pdx2 with homogenous dodecameric Pdx1, we observed the formation of a Pdx complex with slightly higher R_H_ value distribution over time. The obtained suspension did not entirely show a monodispersed Pdx complex; presumably some high-molecular-weight Pdx2 multimers remained in solution 12 h after measurement (Figure 2b and Supplementary Video S2). In addition, a monodispersed Pdx complex solution was obtained after centrifuging suspensions of homogenous Pdx1 and nonhomogeneous oligomeric Pdx2 at 10,000× *g* for 10–15 min, as shown in Supplementary Figure S1e.

### 2.4. SAXS Analysis

**Figure 3:**
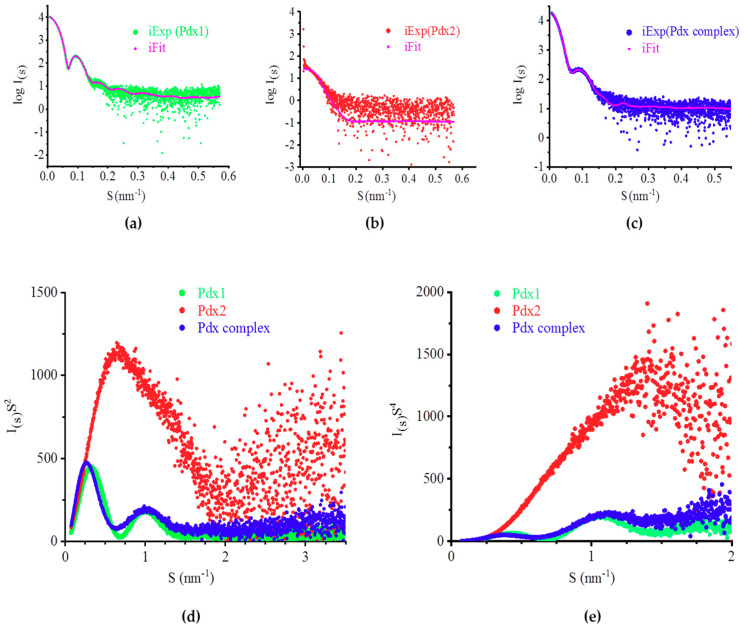
Processed solution scattering intensity pattern of (**a**) Pdx1 (green), (**b**) Pdx2 (red), and (**c**) the Pdx complex (blue) in arbitrary intensity units. Their fits with selected homolog structures are shown in magenta (Pdx1: PDB ID 4ADU; Pdx2: PDB ID 2ABW; Pdx complex: PDB ID 4ADS). Data were processed and evaluated by applying the programs CRYSOL and SASREF; (**d**) dimensionless Kratky plots indicate globular and compact structures for Pdx1 and the Pdx complex and a more flexible structure for Pdx2 (Pdx1 is shown in green, Pdx2 in red, and the Pdx1–Pdx2 complex in blue); (**e**) Porod–Debye plots of the X-ray solution scattering (SAXS) data including the color-coding used in (**d**).

To further analyze the molecular structure, dynamicity, and oligomerization of the Pdx complex and its subunits in solution, monodispersed solutions of Pdx1, Pdx2, and the Pdx complex were subjected to SAXS experiments; their respective averaged scattering intensity profiles are displayed in Figure 3a–c. Guinier analysis using the Guinier approximation and radius of gyration (R_G_) as determined by AUTOGNOM provided R_G_ values for Pdx1, Pdx2, and their complex, which are summarized in Table 1.

The distance distribution function *P(r)* indicated a maximum diameter (*D*_max_) of 15 nm and a nearly globular shape for Pdx1, a *D*_max_ of 11.3 nm, and a rod-shaped structure for Pdx2, as well as *D*_max_ of 22.2 nm for the Pdx complex with a more spherical but slightly extended structure (Supplementary Figure S2 and Table 1). Kratky plots (I_(S)_S^2^ versus S) obtained from the scattering data were used to verify the flexibility of the proteins. Based on the Kratky plots of the scattering data, Pdx1 and the Pdx complex represented rigid and compactly folded particles (Figure 3d). In contrast, the Kratky plot of Pdx2 indicated a significant intrinsic flexibility [26] (Figure 3d). Porod–Debye plots (I_(S)_S^4^ versus S) of the scattering data of the three proteins showed plateaus, indicating that Pdx1 and the Pdx complex lack disordered regions, whereas Pdx2 is more flexible in solution (Figure 3e). The shape factor (ρ = R_G_/R_H_) [27] gave a value of 0.69 for Pdx1 and 0.71 for the Pdx complex, indicating both as nearly spherical globular particles, whereas Pdx2 exhibited values ranging from 0.931 to 1.18, indicative of a flexible ellipsoidal structure [28]. All experimental parameters are summarized in Table 1.

**Figure 4:**
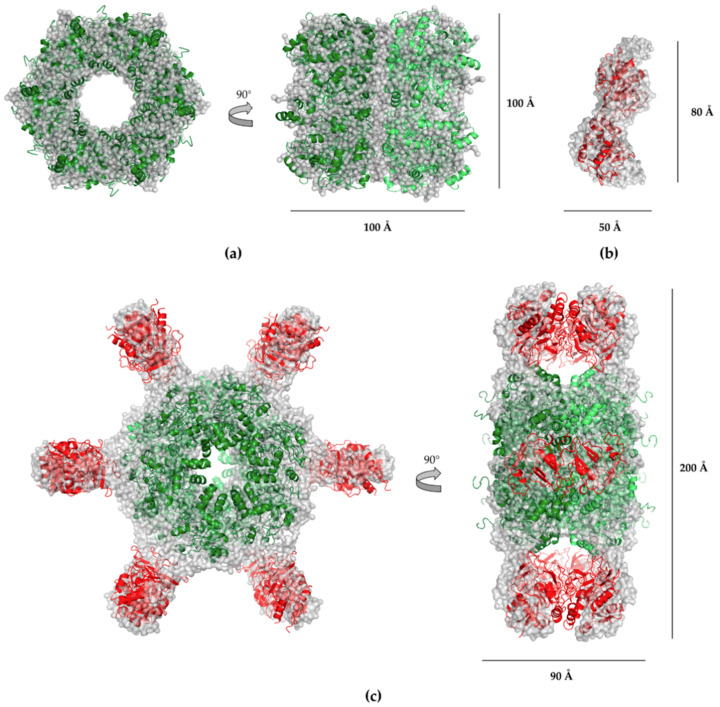
Ab initio GASBOR models composed of light grey-colored, chain-like dummy surface models superimposed with the rigid body SASREF models shown in cartoon representation. (**a**) Dodecameric Pdx1 rigid body model, i.e., one hexamer in green and one in lime color, considering a P6 symmetry superimposed with its ab initio model; (**b**) P1 symmetry for dimeric Pdx2 in red fitted to its ab initio model; (**c**) ab initio model of the Pdx1–Pdx2 (12:12) complex possessing P6 symmetry with two subunits: two hexameric ring-shaped inner cores consisting of Pdx1 (green and lime color) and a total of 12 Pdx2 molecules (red) displaying a hexameric symmetry around the outside surface of both stacked Pdx1 hexamers corresponding to the obtained ab initio model. The ab initio model is superimposed well with the rigid body Pdx complex.

The obtained scattering amplitudes for Pdx proteins were processed by using the software PRIMUS [29] and were compared to homologous crystal structures of *P. falciparum* and *P. berghei* with the following pdb codes using CRYSOL: Pdx1, 4ADU; Pdx complex, 4ADS, and Pdx2, 2ABW [30] (Figure 3a–c). The experimental scattering curves, when compared with the calculated scattering amplitudes, confirmed structural similarities to the respective homologous three-dimensional structures over a wide range of angles. 3D models for Pdx1, Pdx2, and the Pdx complex were constructed using a homology modeling approach [31,32], where Pdx1 was modeled as a dodecamer, Pdx2 in monomeric form, and the Pdx complex as 24-mer. A 3D model for Pdx1 was calculated based on *Pb*Pdx1 (pdb code: 4ADU) [22] for the Pdx complex based on the chimeric complex (4ADS) [22] and for Pdx2 based on the pdb entry 2ABW [19]. The reported crystal structures of Pdx proteins from *P. falciparum* and *P. berghei* showed sequence identities of 88% and 85% for Pdx1, respectively, and 75% and 69% for Pdx2, respectively, compared to the *P. vivax* counterparts [33]. The calculated 3D models of Pdx1, Pdx2, and the Pdx complex were superimposed by applying the program Pymol [34] with the ab-initio-built GASBOR models individually and fitted well with dummy sphere models. Pymol superimpositions were performed for Pdx1 (dodecamer) with its ab initio built model, and its fit indicated Pdx1 to be a dodecamer. Further, the SAXSMoW data resulted in a molecular mass of 418.2 kDa for Pdx1 (theoretical mass value is 417.3 kDa) [35]. For the Pdx complex, a molecular mass estimation of the SAXS data indicated 735.8 kDa, which was very close to the theoretically calculated mass of 730.5 kDa. The obtained rigid body models were separately superimposed with the ab initio models of Pdx1 (Figure 4a), Pdx2 (Figure 4b), and the Pdx complex (Figure 4c), indicating an appropriate fit for Pdx1 and the Pdx complex. Due to reversible oligomerization behavior and the variable oligomeric states of Pdx2, rigid body models were built and calculated in regard to monomeric, dimeric, and elongated trimeric forms. The dimeric rigid body model fit best when superimposed with the ab initio models (Figure 4b), considering an additional significant contribution of trimeric Pdx2 to the scattering intensity pattern. A significant contribution of the trimer was also indicated by SAXSMoW data, with a corresponding molecular mass value of approximately 67 kDa using the Pdx2 scattering profile, whereas the theoretical calculated mass of a Pdx2 monomer was 26.1 kDa. All ab initio and rigid model superpositions were performed by applying Pymol [34].

### 2.5. Electron Microscopy (EM) Analysis

**Figure 5:**
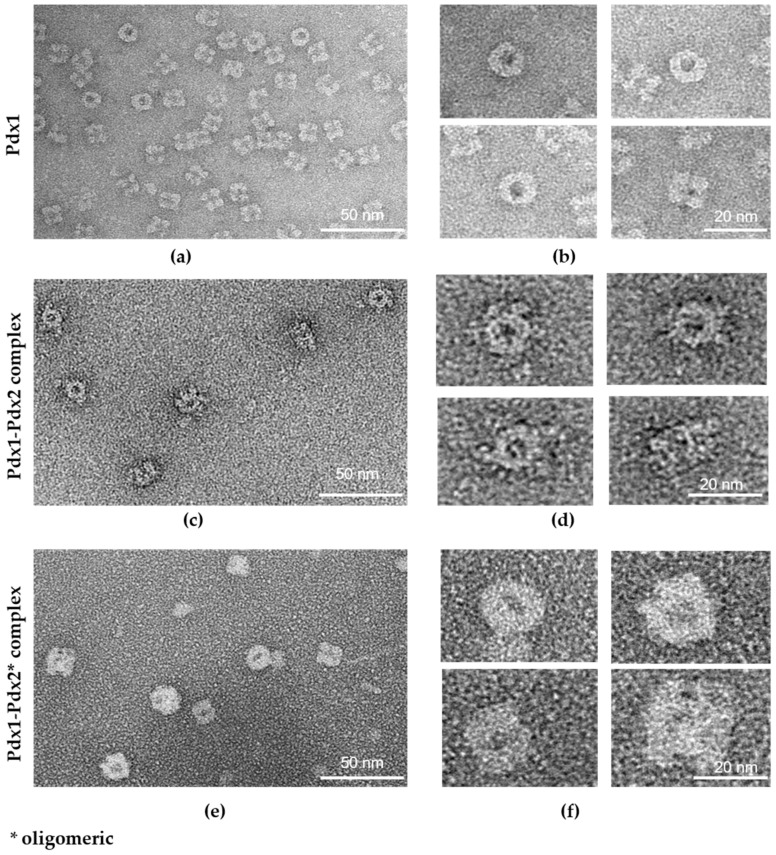
Transmission electron micrographs of negatively stained *P. vivax* pyridoxal phosphate (PLP) synthase proteins. Images on the right in (**b**,**d**,**f**), alongside a 20 nm scale bar, show a zoom-in of a representative class of averaged sample pictures shown on the left in (**a**,**c**,**e**). (**a**,**b**) Dodecameric Pdx1, top view, and side view with random orientations of the particles; (**c**,**d**) dodecameric Pdx1 in complex with Pdx2; for complex formation, a monomeric Pdx2 solution was provided. Pdx complex particles generally only with partially bound Pdx2 were observed and saturated (12:12) Pdx complex was rarely observed; (**e**,**f**) dodecameric Pdx1 in complex with Pdx2 oligomers. The Pdx complex was predominantly saturated with Pdx2, showing a slightly larger dimension than those observed for the Pdx complexes shown in (**c**,**d)**, demonstrating the larger hydrodynamic radius of the corresponding Pdx complex observed by DLS (Figure 2b). * oligomeric Pdx2.

The dodecameric *P. vivax* Pdx1 structure was further analyzed by negative-stain EM analysis, and the resulting images are shown in Figure 5a,b, thereby validating the characteristic Pdx1 dodecameric assembly made up of two hexameric rings.

The Pdx complex was also analyzed by electron microscopy, showing a partial saturation of the ring shaped Pdx1 dodecamer with Pdx2 domains (Figure 5c,d) and confirming the reported data that the attachment of Pdx2 to the dodecameric core of Pdx1 takes place gradually [22]. Analysis of electron microscopy figures showed that Pdx1 and Pdx2 assemble together transiently, and on average, a few Pdx2 subunits occupy Pdx1 dodecamers without preference for a distinct association pattern. Further, the reversible oligomerization behavior of Pdx2 was analyzed by EM to confirm the observed hydrodynamic radius shift recorded by DLS after mixing dodecameric Pdx1 with monomeric in one experiment and oligomeric Pdx2 in another experiment. The obtained EM micrographs confirmed the DLS data and showed that Pdx1 was complexed with Pdx2. As seen in Figure 5d, monomeric Pdx2 bound to dodecameric Pdx1; however, not all available vacancies were filled, whereas the binding of oligomeric Pdx2 to the structured Pdx1 12-mer resulted in a slightly asymmetric ring morphology of the Pdx complex particles, as shown in Figure 5e,f. The Pdx complex particles shown in Figure 5f were also slightly larger than those shown in Figure 5d.

## 3. Discussion

Despite some coordinated efforts by the WHO and national health organizations, malaria still exists as an endemic disease in Africa, South America, and Asia [2]. Drug resistance for *P. falciparum, P. vivax,* and *P. malariae* is recognized mainly as a result of long-term overuse of antimalarial antibiotics [36]. This necessitates the identification of new antimalarial drug targets.

*Plasmodium* species possess the plasmodial PLP synthase, acting as a functional vitamin B6 biosynthesis pathway [9]. The class I GATase (glutamine amidotransferase) pathway consists of the synthase subunit (Pdx1) and the glutaminase subunit (Pdx2). Pdx2 produces ammonia from glutamine, which passes through a hydrophobic structural region named the ammonia tunnel and reaches the active site of Pdx1, where ammonia in the presence of intermediates from pentose and triose forms PLP (vitamin B6) [19] with their dissociation constant (KD) values reported from plasmodial Pdx proteins [12]. Interestingly, Pdx1 and Pdx2 require each other for activation and substrate utilization, highlighting the ammonia tunnel entrance as an interesting target region for drug design and drug discovery investigations due to its presence within the protein–protein (Pdx1–Pdx2) interface.

Pdx1 and Pdx2 are expressed throughout the erythrocytic cycle of *Plasmodium*, and both are cytosolic proteins able to interact and form the structural distinct Pdx complex, thereby generating vitamin B6 during all developmental stages of the parasite. The absence of this metabolic pathway in humans makes this vitamin B6 biosynthesis pathway an interesting target for novel antimalarial drugs [37]. In this context, it is important to understand the structure and dynamics of Pdx complex assembly, as well as to analyze the structures of the individual components, Pdx1 and Pdx2, which assemble to form the 12:12 Pdx complex. It was reported previously that *B. subtilis* Pdx1 (*Bs*Pdx1) exists in an equilibrium of hexamers and dodecamers [15]. For *G. stearothermophilus*, Pdx1 analytical ultra-centrifugation (AUC) studies showed a hexamer–dodecamer in solution, whereas dodecamer was present in the crystalline form [14]. Further, the eukaryote *S. cerevisiae* Pdx1 (*Sc*Pdx1) was reported to be hexameric in solution [17] and the eukaryotic *Pf*Pdx1 was reported to exist in a dodecameric form in solution [22]. In contrast to bacterial homologs and the eukaryote *S. cerevisiae*, *Pf*Pdx1 even forms higher-order oligomers/fibers [22].

**Figure 6:**
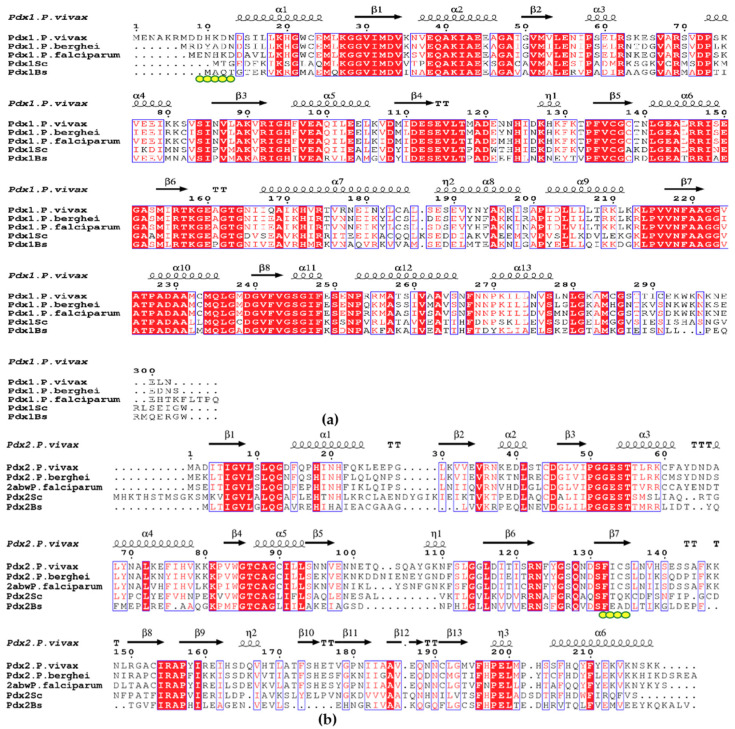
(**a**) Alignment of Pdx1 amino acid sequences from *P. vivax* (A5K247) *P. berghei* (Q4Z0E8), *P. falciparum* (C6KT50), *S. cerevisiae* (Q03148), and *B. subtilis* (P37527); (**b**) Alignment of Pdx2 amino acid sequences from *P. vivax* (A0A1G4GXI4) *P. berghei* (Q4PJX5), *P. falciparum* (Q8IIK4), *S. cerevisiae* (Q03144), and *B. subtilis* (P37528). The active site residue of Pdx2 H-196 is mutated in *Pf*Pdx2 to N. The red boxes with white lettering show strict identity, red letters show similarity in the region between amino acid groups, and black letters show that regions are not conserved, with TT being a strict β turn and TTT a strict α turn. The Pdx1–Pdx2 contact surface involves many backbone interactions that are very much conserved in the three-dimensional structure but are not strictly conserved in the primary structure of Pdx2. Pivotal in the complex formation is the nonconserved region between β-5 and β-6 (η1) and the sequence region between β6 and β7 of the glutaminase. The yellow dots in Pdx1 mark the βN region, which interacts with the region (131–134) in the Pdx2 involved in β-completion and complex stabilization in bacterial complexes. This β-completion is not reported in plasmodial species [22], but the elongated N-terminus in *Pv*Pdx1 (which is absent in other plasmodial homologs) may have a role in complex stabilization. The sequence alignment was prepared by Espript3 [42].

In terms of drug discovery investigations to treat malarial infection, we investigated *P. vivax* Pdx proteins by applying complementary biophysical techniques and compared our results to selected eukaryotic Pdx proteins (sequence comparison in Figure 6a,b). *P. vivax* Pdx1 (*Pv*Pdx1) was investigated by SEC and DLS studies, showing a dodecameric state which is stable for at least 20 days in solution (Figure 1a,f). SAXS data (Figure 4a) and EM investigations (Figure 5a,b) confirmed the dodecameric state of Pdx1 in solution and revealed a low-resolution structure. The sequence alignment (Figure 6a), of residues I-166 to L-211 showed that helices α6, α6′, and α6′′, which stabilize the dodecamer, are highly conserved among the plasmodial species and significant variations are only observed at the N- and C- terminus [38]. It can be assumed that the nonconserved and probably flexible C-terminus of Pdx1, which is absent in other plasmodial homologs except for *P. falciparum*, is prone to degradation [22], and presumably also involved in the fiber formation observed in *Pf*Pdx1.

Higher-order oligomers and fiber formation were also reported for the *Pf*Pdx complex upon storage at 4 °C, as observed by analytical SEC and EM analysis [22]. For the *P. vivax* Pdx complex, we showed by applying SEC, DLS, SAXS, and EM that the complex was stable in solution without observing higher-order oligomers over time. Further, no significant concentration-dependent oligomerization for Pdx1 and the Pdx complex at concentrations ranging from 1 to 20 mg mL^−1^ was identified.

*Pf*Pdx2 was previously reported to be monomeric in solution and dimeric in the crystal lattice [19]. In contrast, we found that *Pv*Pdx2 was predominantly monomeric in solution, however a fraction of oligomers were identified in solution by DLS and SAXS. Our CD results indicated approximately 64% β sheets and coils, thereby showing that Pdx2 is prone to some oligomerization as increased β sheet content increases the potential for oligomerization [39]. TANGO, a statistical mechanics algorithm based on the physicochemical principles of β-sheet formation to detect protein oligomerization, suggested that the regions 4–10, 30–35, 84–94, and 182–186 of *Pv*Pdx2, which form β sheets, support oligomerization [40].

We further report for the first time that the oligomerization of Pdx2 is concentration-dependent and reversible upon interacting with dodecameric Pdx1 (Figure 2b and Supplementary Figure S1). The reversible oligomerization of Pdx2 toward complex formation with Pdx1 was analyzed by time-resolved DLS and confirmed by EM. EM analysis showed the Pdx complex formation after oligomeric Pdx2 interacted with dodecameric Pdx1.

For both plasmodial and bacterial PLP synthases, Pdx2 is active only in the presence of Pdx1 [19,41]. The interaction between the two proteins (Pdx1 and Pdx2) is mediated by the conserved helix αN of Pdx1 (Figure 6a) [12]. This helix is slightly longer in *P. vivax* than observed for other plasmodial homologs, which presumably results in different affinity between Pdx1 and Pdx2 [7] and also different stability of dodecameric Pdx1 and the Pdx complex.

After analyzing the PLP synthase proteins and the complex formation by applying different biophysical techniques, it can be concluded that, in comparison to other plasmodial homologs, Pdx1 and the Pdx complex from *P. vivax* are more stable in solution than their homologs. However, the Pdx complex can show variations in terms of attachment of monomeric and oligomeric Pdx2.

## 4. Materials and Methods

### 4.1. Expression and Purification of P. vivax Pdx1, Pdx2, and Complex Formation

Genes of Pdx1 and Pdx2 with restriction sites recognized by *NdeI* and *XhoI* were cloned into the expression vector pET31b(+) (BioCat GmbH, Heidelberg, Germany) separately. Cells of *Escherichia coli* strain BL21-CodonPlus-(DE3)-RIL were transformed with the expression vector, grown at 37 °C, and induced by 0.5 mM IPTG. The filtered supernatant from cells lysed by sonication was applied to a two-step purification protocol, i.e., Ni-NTA affinity chromatography and SEC. Pure Pdx1 and Pdx2 were obtained using a Superdex 200 column (26/60, GE Healthcare, Uppsala, Sweden) and mixed in a 1:1 molar ratio and incubated overnight with 10 mM l-glutamine. The resulting protein complex was finally purified by a Superose-6-increase column (10/300, GE Healthcare, Uppsala, Sweden).

### 4.2. Dynamic Light Scattering (DLS) and Circular Dichroism (CD) Investigations

For DLS analysis, sample solutions were measured in a quartz cuvette using the Spectroscatter-301 (Xtal Concept, Hamburg, Germany) applying a laser wavelength of 660 nm. The scattered light was collected at a fixed angle of 90°. The autocorrelation function was processed by applying the Stokes–Einstein equation and the particle size distributions were calculated from the translational diffusion coefficient.

To evaluate the secondary structure content and to score the overall folding of Pdx1, Pdx2, and the Pdx complex, CD spectroscopy investigations were performed. CD spectra of the samples were recorded at 20 °C by utilizing a J-815 CD spectrometer (Jasco, UK). The mean molar ellipticity (deg cm^2^ dmol^−1^) was plotted against the wavelength applying the Spectra Manager^TM^ software, and the secondary structure contents were determined with the Spectra Manager^TM^ software [23].

### 4.3. Solution Small Angle X-Ray Scattering (SAXS)

Pdx1, Pdx2, and the Pdx complex were analyzed by SAXS. Data were collected at the EMBL beamline P12 (PETRA III, DESY, Hamburg, Germany). For complex formation, pure Pdx1 and Pdx2 were mixed in a 1:1 ratio and incubated overnight. Prior to SAXS data collection, the complex was purified by a Superose-6-increase column (10/300, GE Healthcare). Pdx1 and the Pdx complex were investigated at concentrations of 1, 2, and 5 mg mL^−1^ by applying an automated robotic sample changer and a Dectris 2D photon-counting detector (PILATUS-6M) with 3.1 m sample to detector distance. Considering the oligomerization tendency of Pdx2, online size-exclusion chromatography was applied prior to SAXS data collection (SEC–SAXS) by utilizing 7 mg mL^−1^ Pdx2 and a Superdex-75-increase column (5/150, GE Healthcare), which was pre-equilibrated with a buffer of 20 mM Tris-HCl (pH 8.0), 200 mM NaCl, 1 mM EDTA, 2 mM DTT, and 3% glycerol. The obtained eluted sample solution was directly subjected to X-ray scattering data collection.

Scattering data for all three samples were collected, integrated, and averaged by applying the SasTool software (EMBL, Hamburg, Germany) [43]. Guinier analysis and radius of gyration (R_G_) values were calculated using PRIMUS [29]. The pair distribution functions *P(r)* and forward scattering intensities *I*(0) were processed with GNOM [44] and PRIMUS [29]. Obtained data were compared with the values provided by SAXSMoW [35]. Kratky plots (I_(S)_S^2^ versus S) and Porod–Debye plots (I_(S)_S^4^ versus S) were generated, as described previously [45], and molecular mass (MM) data were obtained from SAXSMoW [35]. Low-resolution ab initio models were generated using the programs DAMMIN and GASBOR, considering the symmetry option P1 for Pdx2 and symmetry P6 for Pdx1 and the Pdx complex [46,47]. Rigid body models were constructed applying the program SASREF [48] considering the same symmetry options as those used for the dummy models. Theoretical scattering curves of homologous structures deposited at the protein data bank were calculated, compared, and fitted to the experimental data using CRYSOL [30].

### 4.4. Electron Microscopy (EM) Analysis

For EM investigations, sample solutions with concentrations between 25 and 30 µg mL^−1^ of Pdx1, Pdx1 in complex with Pdx2 monomers, and Pdx1 in complex with Pdx2 oligomers in 20 mM Tris pH 8.0 containing 200 mM NaCl and 5 mM MTG (monothioglycerol) were stained with 2% uranyl acetate. Specimens were prepared for EM investigations using a conventional negative staining procedure [49]. Sample solution drops of 4 µL were adsorbed to a glow-discharged, carbon-coated, copper grid, washed with two drops of deionized water, and stained with two drops of freshly prepared 2% uranyl acetate solution. Micrographs were recorded with a Talos L120C (Thermo Fisher Scientific-FEI) electron microscope at 120 kV accelerating voltage and a LaB6 source filament with a CETA camera. Images were taken using a magnification of 71,000× to 92,000×.

## 5. Conclusions

We presented a detailed analysis of the *P. vivax* Pdx proteins, Pdx1 and Pdx2 in solution, that form the pyridoxal phosphate (PLP) biosynthesis enzyme complex and provided structural details about the *P. vivax* Pdx1 dodecamer, the oligomerization behavior of Pdx2, and the dynamics of Pdx complex formation. Our investigations applying complementary bioanalytical techniques showed that purified *P.vivax* Pdx1 formed a stable dodecamer in solution and that purified Pdx2 is present as a monomer but forms different oligomers in solution over time. Time-resolved DLS experiments revealed the dynamics of Pdx complex formation and completion in hours by monitoring a solution of Pdx1 dodecamer and adding Pdx2. In contrast to Pdx1, the PLP synthase domain of the pyridoxal phosphate (PLP) biosynthesis complex, purified Pdx2 could be detected in different oligomeric states; interestingly, however, the complex exhibited a distinct tendency to specifically interact with Pdx1, resulting in a hetero-oligomeric Pdx complex irrespective of the monomeric or oligomeric state of Pdx2 in solution. These results highlight that the Pdx1 dodecamer is the essential and stable subunit in the Pdx complex acting as an anchor, and Pdx2 can bind to the Pdx1 dodecamer upon availability.

## Figures and Tables

**Table 1:** SAXS data collection and analysis parameters.

	Pdx1	Pdx2	Pdx Complex
Data collection parameters			
X-ray source	PETRA III; EMBL beamline P12		
Wavelength (nm)	0.124		
Detector distance (m)	3.1		
Temperature (K)	293	283	293
Structural parameters			
*I*(0) (*P(r)* function)	10,720 ± 5.93	9161 ± 41.47	19380 ± 23.04
*I*(0) (Guinier/AutoR_G)_	10,727.20 ± 11.61	9307.96 ± 101.56	19,658.30 ± 28.99
*I*(0) (SAXSMoW)	10,751.50	33.64	19,592.77
R_G_ (nm) (*P(r)* function)	5.00 ± 0.01	3.26 ± 0.02	6.83 ± 0.01
R_G_ (nm) (Guinier/AutoR_G_)	5.05 ± 0.06	3.24 ± 1.36	6.95 ± 0.11
R_G_ (nm) (based on SAXSMoW)	5.07	3.52	6.89
qR_G_ limit (from Guinier/AutoR_G_)	1.12	1.29	1.15
qR_G_ limit (from SAXSMoW)	1.29	1.30	1.29
*D*_max_ (nm) (from *P(r)* function)	15.0	11.3	22.2
Porod volume estimate (nm)	609.54	75.88	1733.18
P = R_G_/R_H_	0.69	0.93–1.18	0.71
Molecular mass determination (kDa)			
Molecular mass (SAXSMoW)	418.2	67.0	735.8
Molecular mass (amino acids sequence)	417.3	26.1	730.3
Oligomeric state	Dodecamer	Oligomer	24-mer

## Supplementary Materials

Supplementary materials can be found at https://www.mdpi.com/1422-0067/21/17/5971/s1.

## Abbreviations

AUC	Analytical ultra-centrifugation
BZU	Bahauddin zakariya university
CD	Circular dichroism
CSSB	left for structural systems biology
DESY	Deutsches electronen-synchrotron
DLS	Dynamic light scattering
Dmax	Maximum diameter
EM	Electron microscopy
EMBL	European molecular biology laboratory
HEC	Higher education commission
I(s)	Scattering intensity
MM	Molecular masses
MTG	Monothioglycerol
Ni-NTA	Nickel nitrilotriacetic acid
NTA	Nanoparticle tracking analysis
PAGE	Polyacrylamide gel electrophoresis
PDB	Protein data bank
Pdx	Pyridoxal
Pr	Distance distribution function
PLP	Pyridoxal phosphate
Pv	*Plasmodium vivax*
Pf	*Plasmodium falciparum*
R_G_	Radius of gyration
R_H_	Hydrodynamic radius
RMS	Root mean square deviation
SAXS	Small angle x-ray scattering
SDS	Sodium dodecyl sulphate
SEC	Size-exclusion chromatography
ρ	Shape factor
