# Supplementary material for: Solution Structures and Dynamic Assembly of the 24-Meric Plasmodial Pdx1–Pdx2 Complex
Source: Int J Mol Sci. 2020 Aug 19;21(17):5971. doi: 10.3390/ijms21175971 (PMC7504066; doi:10.3390/ijms21175971)
Supplement: Supplementary file 1 [file ijms-21-05971-s001.pptx]

## Slide 1
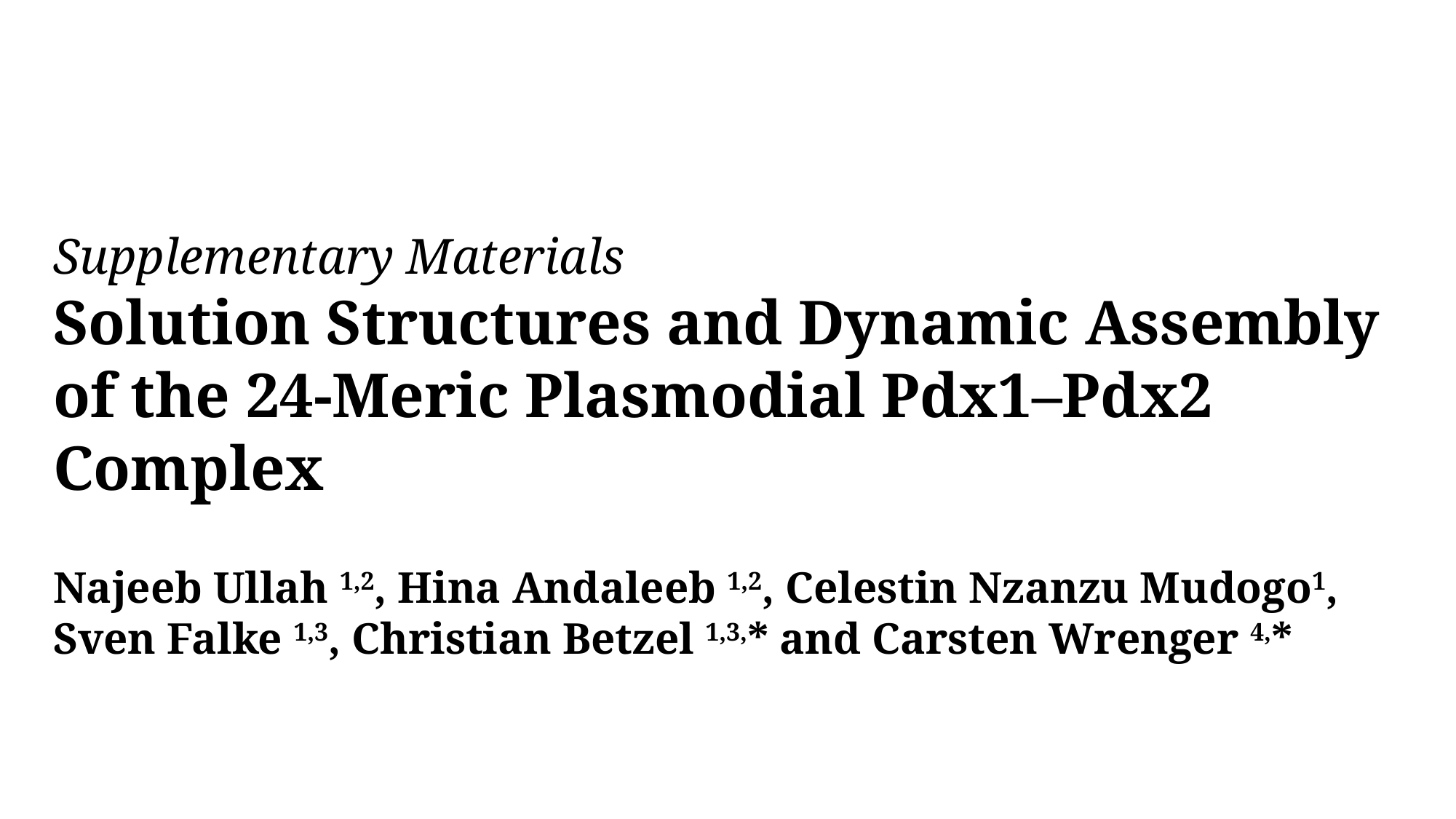

Supplementary Materials
Solution Structures and Dynamic Assembly of the 24-Meric Plasmodial Pdx1–Pdx2 Complex
Najeeb Ullah 1,2, Hina Andaleeb 1,2, Celestin Nzanzu Mudogo1,
Sven Falke 1,3, Christian Betzel 1,3,* and Carsten Wrenger 4,*

## Slide 2
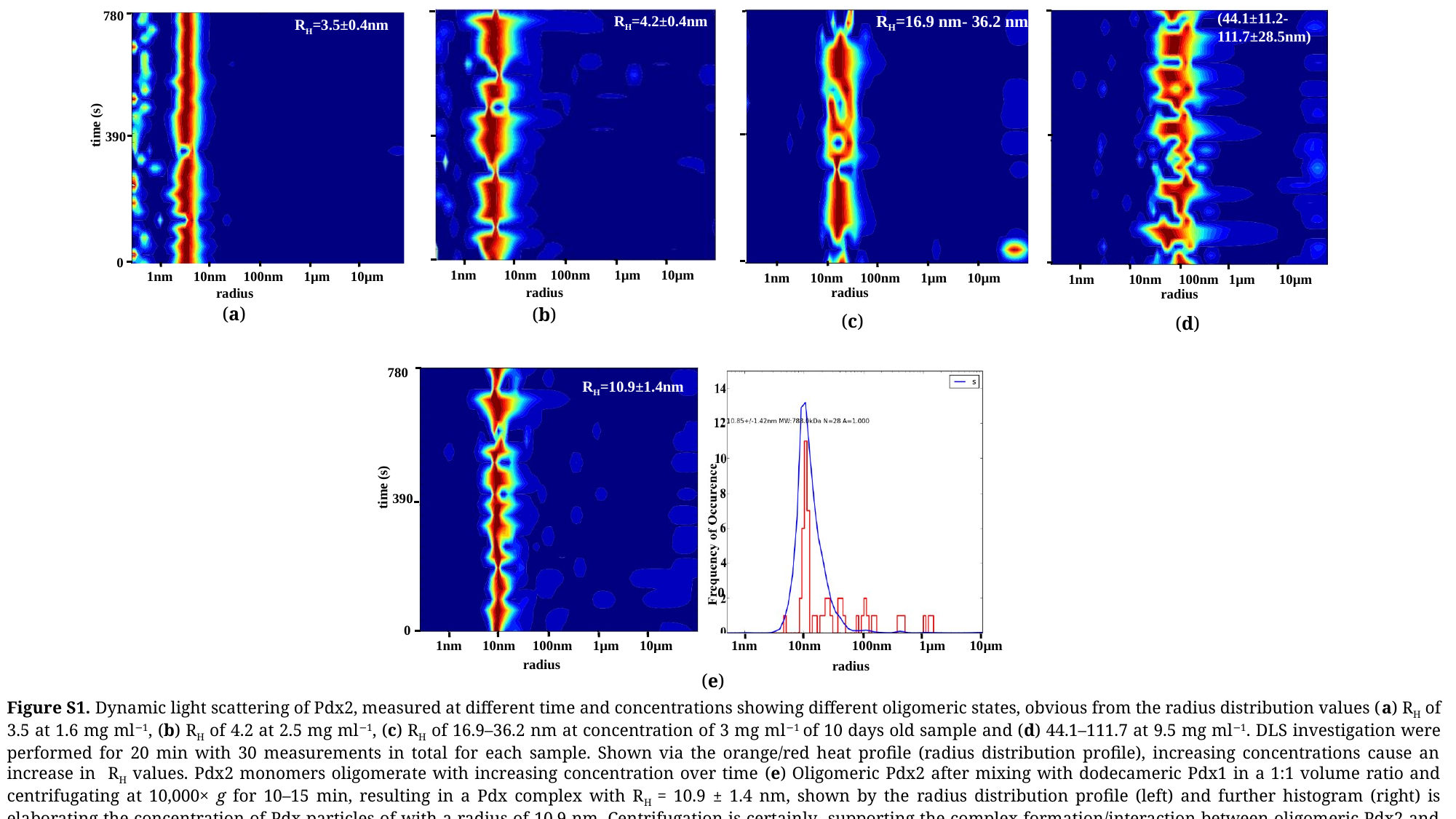

RH=16.9 nm- 36.2 nm
780
(44.1±11.2-
111.7±28.5nm)
RH=4.2±0.4nm
 RH=3.5±0.4nm
time (s)
390
0
 1nm 10nm 100nm 1µm 10µm
1nm 10nm 100nm 1µm 10µm
1nm 10nm 100nm 1µm 10µm
1nm 10nm 100nm 1µm 10µm
radius
radius
radius
radius
780
time (s)
390
0
 RH=10.9±1.4nm
0
1nm 10nm 100nm 1µm 10µm
radius
 1nm 10nm 100nm 1µm 10µm
radius
(b)
(e)
#
(a)
(c)
(d)
Figure S1. Dynamic light scattering of Pdx2, measured at different time and concentrations showing different oligomeric states, obvious from the radius distribution values (a) RH of 3.5 at 1.6 mg ml−1, (b) RH of 4.2 at 2.5 mg ml−1, (c) RH of 16.9–36.2 nm at concentration of 3 mg ml−1 of 10 days old sample and (d) 44.1–111.7 at 9.5 mg ml−1. DLS investigation were performed for 20 min with 30 measurements in total for each sample. Shown via the orange/red heat profile (radius distribution profile), increasing concentrations cause an increase in RH values. Pdx2 monomers oligomerate with increasing concentration over time (e) Oligomeric Pdx2 after mixing with dodecameric Pdx1 in a 1:1 volume ratio and centrifugating at 10,000× g for 10–15 min, resulting in a Pdx complex with RH = 10.9 ± 1.4 nm, shown by the radius distribution profile (left) and further histogram (right) is elaborating the concentration of Pdx particles of with a radius of 10.9 nm. Centrifugation is certainly supporting the complex formation/interaction between oligomeric Pdx2 and dodecameric Pdx1, resulting in a mainly monodisperse solution of Pdx proteins.

## Slide 3
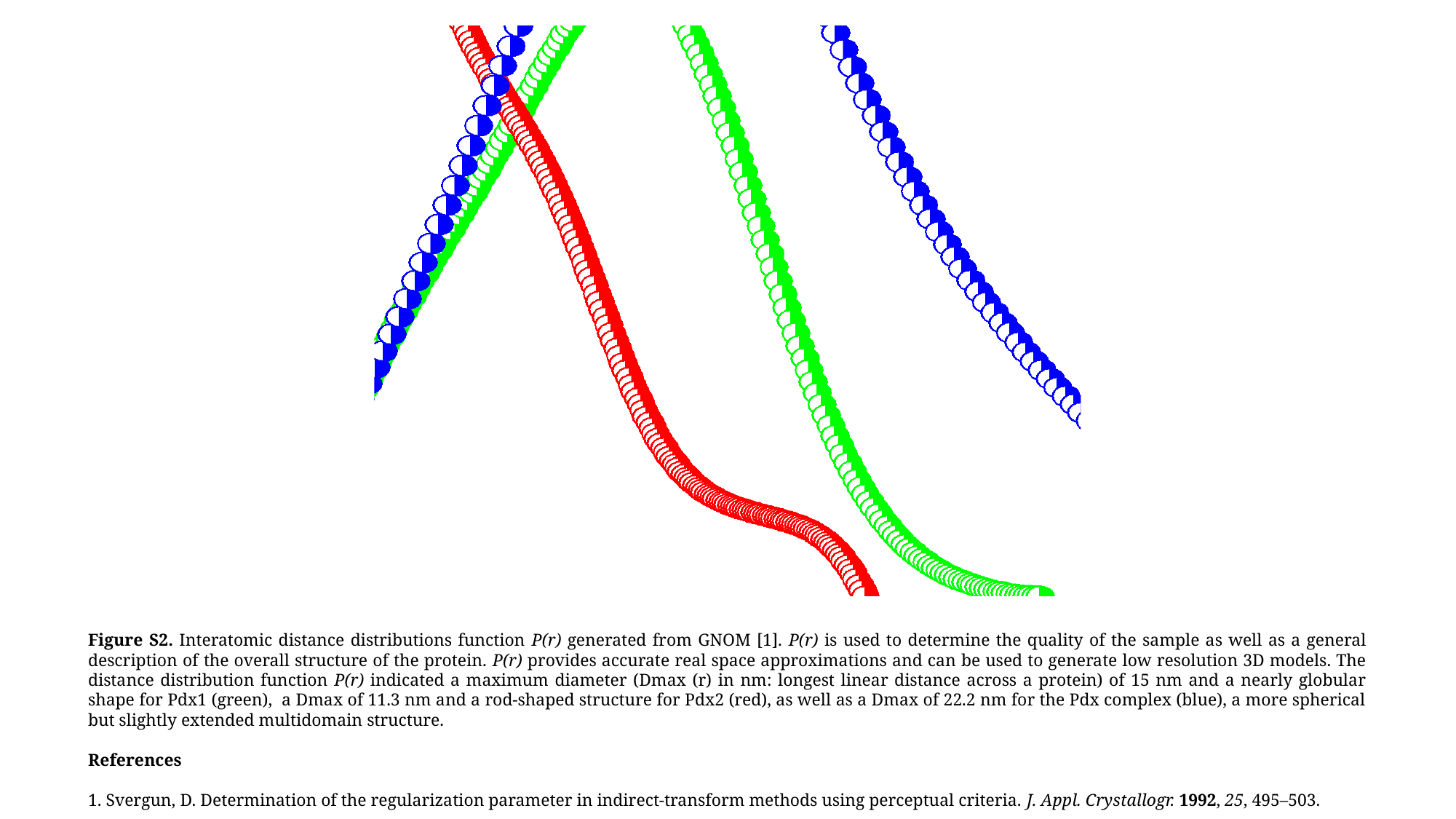

Figure S2. Interatomic distance distributions function P(r) generated from GNOM [1]. P(r) is used to determine the quality of the sample as well as a general description of the overall structure of the protein. P(r) provides accurate real space approximations and can be used to generate low resolution 3D models. The distance distribution function P(r) indicated a maximum diameter (Dmax (r) in nm: longest linear distance across a protein) of 15 nm and a nearly globular shape for Pdx1 (green), a Dmax of 11.3 nm and a rod-shaped structure for Pdx2 (red), as well as a Dmax of 22.2 nm for the Pdx complex (blue), a more spherical but slightly extended multidomain structure.
References
1. Svergun, D. Determination of the regularization parameter in indirect‐transform methods using perceptual criteria. J. Appl. Crystallogr. 1992, 25, 495–503.

## Slide 4
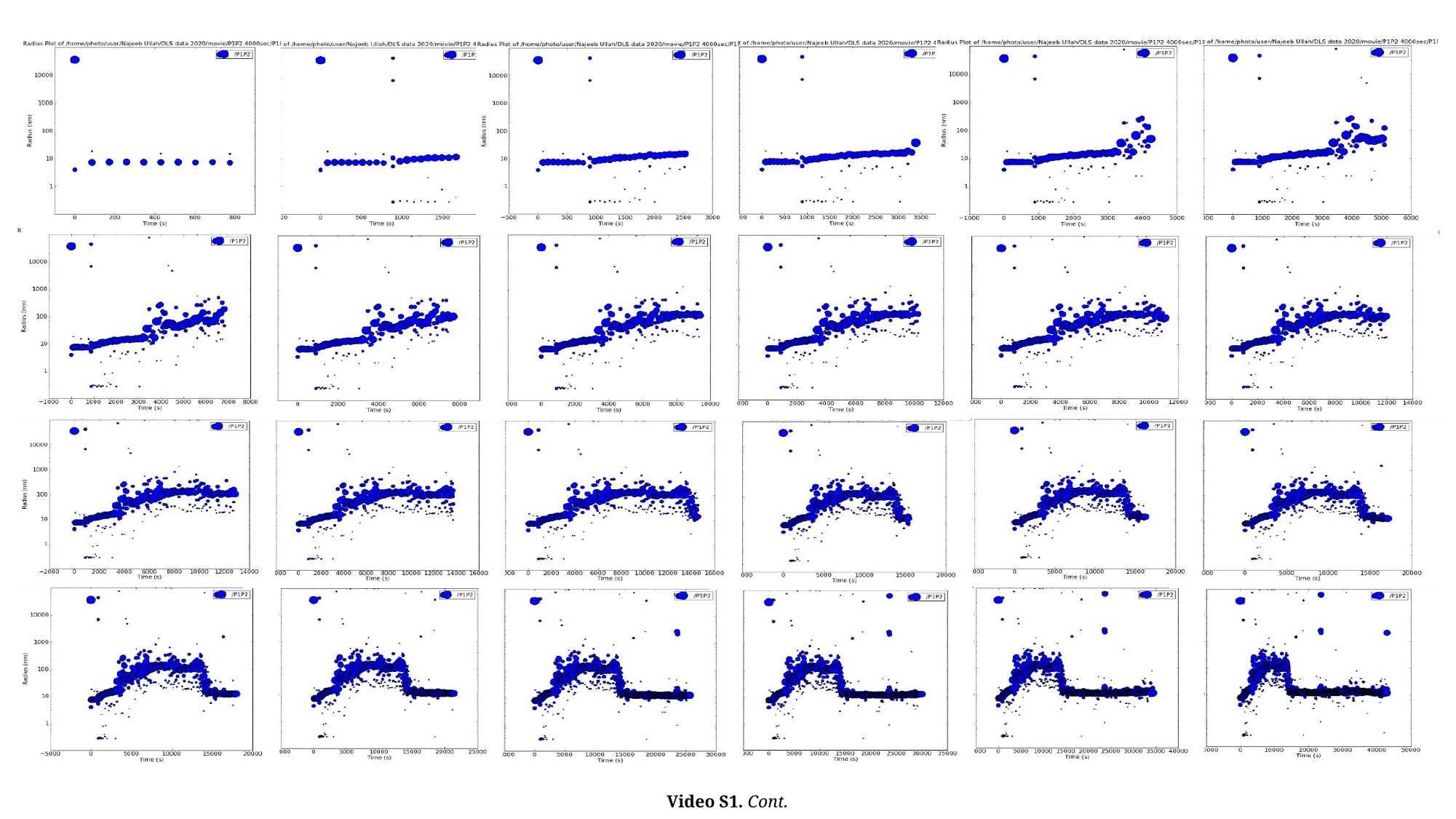

Video S1. Cont.

## Slide 5
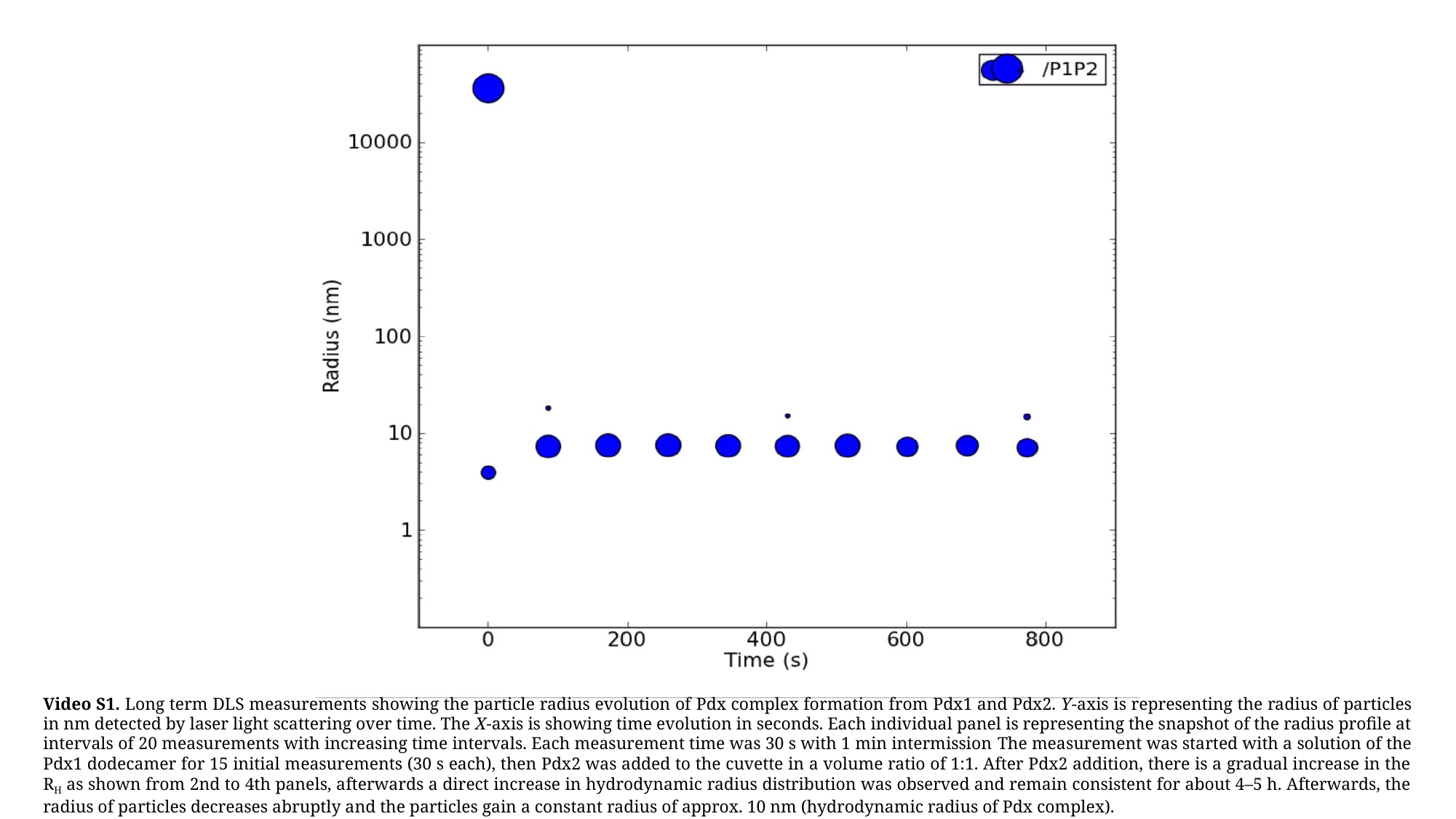

#
Video S1. Long term DLS measurements showing the particle radius evolution of Pdx complex formation from Pdx1 and Pdx2. Y-axis is representing the radius of particles in nm detected by laser light scattering over time. The X-axis is showing time evolution in seconds. Each individual panel is representing the snapshot of the radius profile at intervals of 20 measurements with increasing time intervals. Each measurement time was 30 s with 1 min intermission The measurement was started with a solution of the Pdx1 dodecamer for 15 initial measurements (30 s each), then Pdx2 was added to the cuvette in a volume ratio of 1:1. After Pdx2 addition, there is a gradual increase in the RH as shown from 2nd to 4th panels, afterwards a direct increase in hydrodynamic radius distribution was observed and remain consistent for about 4–5 h. Afterwards, the radius of particles decreases abruptly and the particles gain a constant radius of approx. 10 nm (hydrodynamic radius of Pdx complex).

## Slide 6
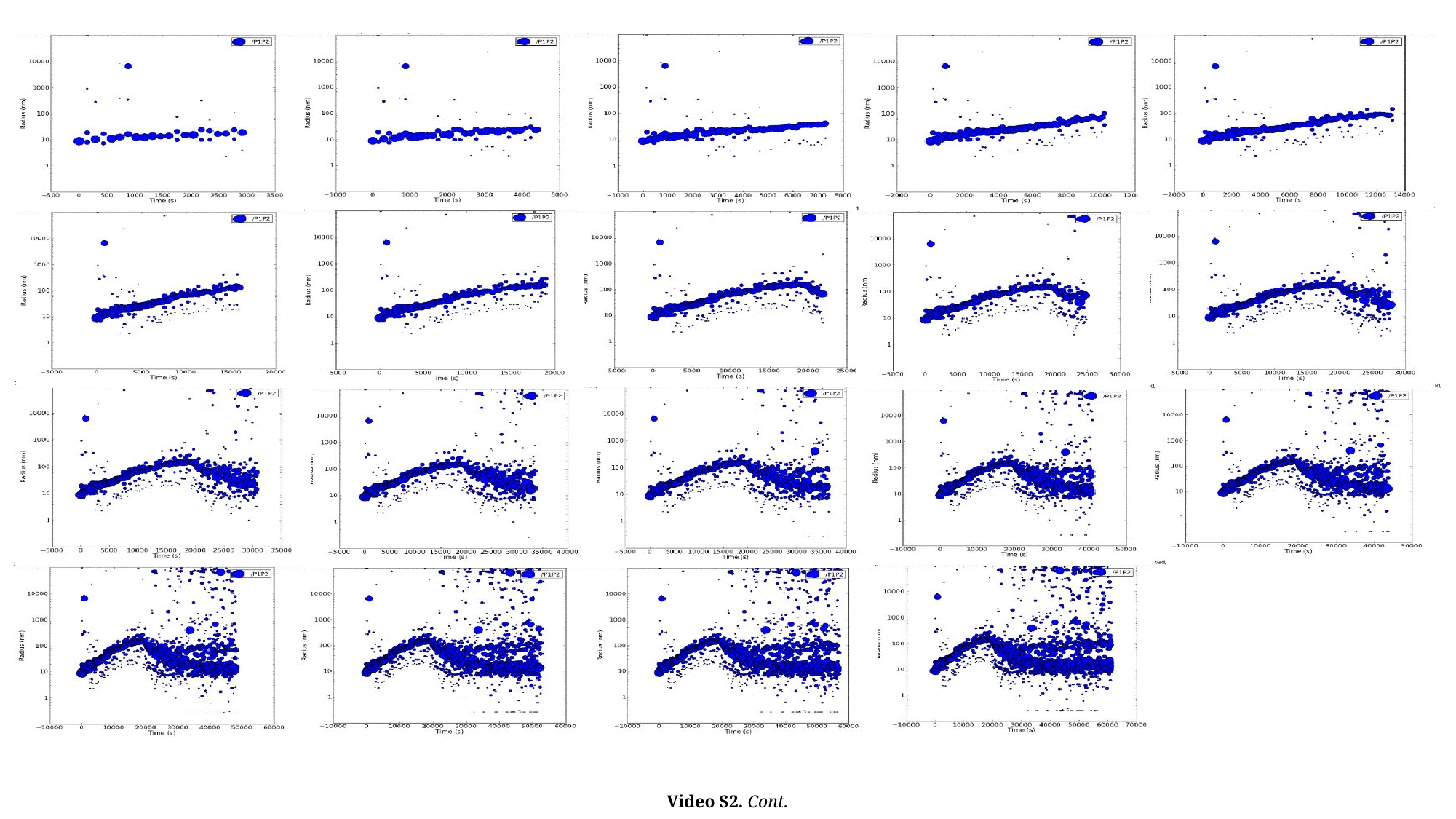

Video S2. Cont.

## Slide 7
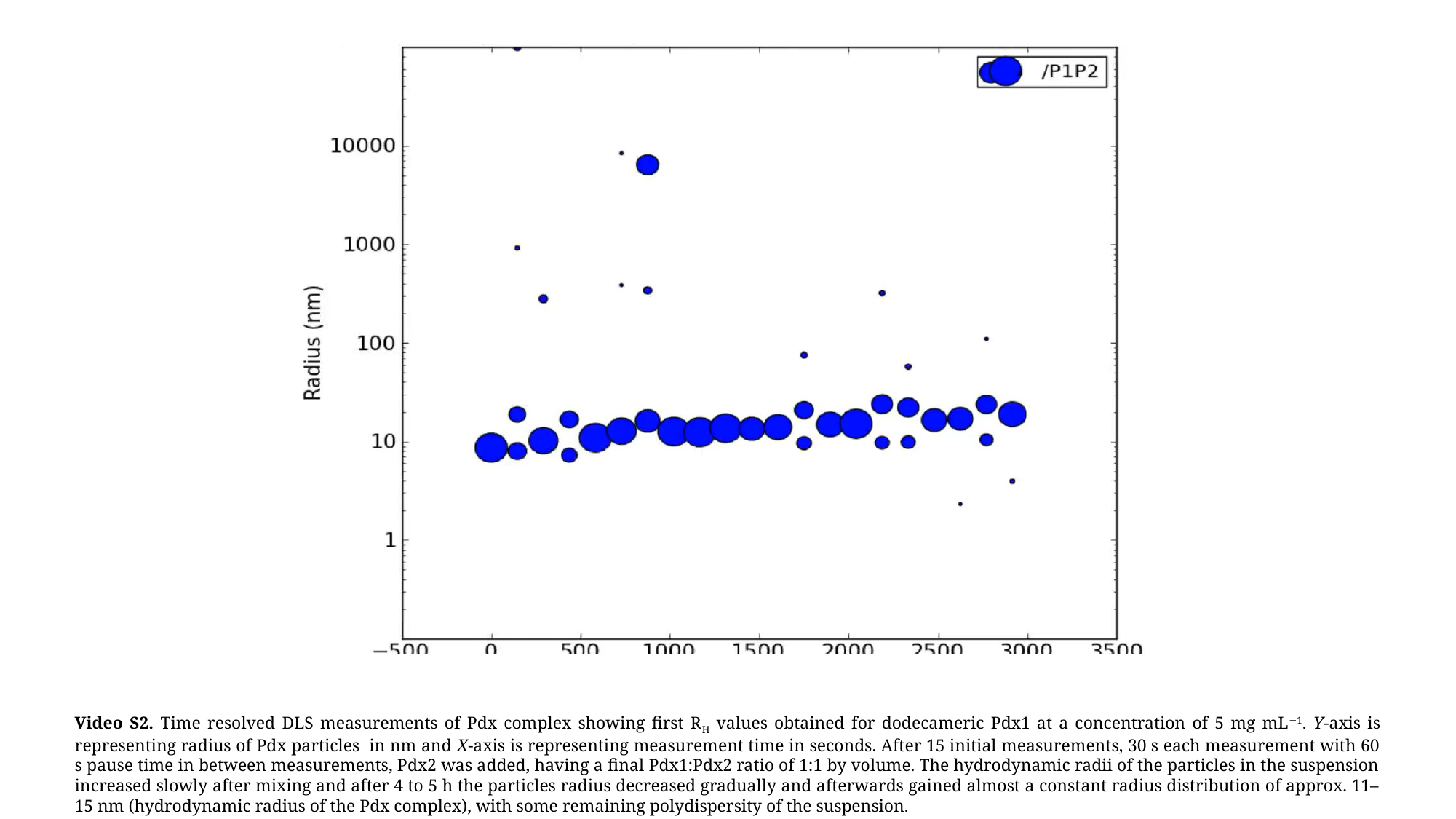

#
Video S2. Time resolved DLS measurements of Pdx complex showing first RH values obtained for dodecameric Pdx1 at a concentration of 5 mg mL−1. Y-axis is representing radius of Pdx particles in nm and X-axis is representing measurement time in seconds. After 15 initial measurements, 30 s each measurement with 60 s pause time in between measurements, Pdx2 was added, having a final Pdx1:Pdx2 ratio of 1:1 by volume. The hydrodynamic radii of the particles in the suspension increased slowly after mixing and after 4 to 5 h the particles radius decreased gradually and afterwards gained almost a constant radius distribution of approx. 11–15 nm (hydrodynamic radius of the Pdx complex), with some remaining polydispersity of the suspension.
